# Investigating Tourists’ Emergency Healthcare Access Barriers: A Systematic Literature Review

**DOI:** 10.3390/healthcare14060761

**Published:** 2026-03-18

**Authors:** Panagiota Peleka, Dimitra-Maria Aggelopoulou, Olga Siskou

**Affiliations:** 1 401 General Military Hospital of Athens, 11525 Athens, Greece; panagiotapeleka@gmail.com; 2 Department of Tourism Studies, University of Piraeus, 18532 Piraeus, Greece; smd2400001@uoa.gr; 3 School of Medicine, National and Kapodistrian University of Athens, 11527 Athens, Greece

**Keywords:** tourists, travelers, emergency medical care, urgent health care, barriers, unmet healthcare needs, accessibility, patient experiences, satisfaction

## Abstract

**Background:** Tourists often travel within their own country or abroad for business, leisure or to receive planned healthcare. However, they are often not prepared for unexpected medical emergencies that occur far from home. Seeking emergency healthcare during travel may pose various barriers and challenges to tourists. **Aims:** This systematic review aimed to identify the challenges and barriers tourists face while seeking emergency healthcare during travel. **Methods:** A comprehensive search was performed in PubMed, Scopus, Web of Science and ScienceDirect from 1st January 1995 to 31 October 2025. The review included studies focusing on tourists who sought emergency healthcare abroad. Due to the methodological heterogeneity of the studies making meta-analysis impossible, a narrative synthesis of the results was conducted. The review protocol was registered with PROSPERO (ID CRD420251156975). **Results:** From 608 initial titles (603 from database searches and 5 additional from similar articles), 10 studies were selected—5 cross-sectional and 5 retrospective. Most (7/10) were conducted in Asian countries, while others were conducted in Europe (1), the U.S.A. (1) and multiple countries (1). The participant number ranged from 37 to 2333. All studies included both genders, apart from one that focused exclusively on pregnant women. The most common challenges identified were language and cultural barriers, limited access to healthcare services in terms of appropriateness and timeliness of care and financial and insurance coverage issues. **Conclusions:** The findings underscore that tourists face multiple barriers when seeking emergency healthcare abroad, resulting in negative tourist travel experiences. Once identified, specific strategies should be adopted to improve accessibility and the overall quality of care for tourists.

## 1. Introduction

Τourists often travel within their own country or abroad for business, leisure or to receive planned healthcare. According to the United Nations World Tourism Organization (UNWTO) [1], approximately 1.4 billion international tourist arrivals were recorded in 2024, indicating an additional rise of between 3% and 5% throughout 2025. The UNWTO attributes this recovery to the “steady” demand from major markets and the rebound of tourism in Asia, which has seen significant growth after the COVID-19 pandemic restrictions.

However, tourists are often unprepared for unexpected medical emergencies encountered far from home. Although some health issues may be mild and resolve spontaneously, medical problems among travelers are not uncommon. Researchers have previously found that between 10,1% and 44,8% of travelers experience illness during international trips [2,3,4,5], but this percentage can rise to nearly 70% when visiting developing countries [6]. According to reports from the Bureau of Epidemiology, Ministry of Public Health of Thailand, the prevalence of the reported health problems was 2.1 per 1000 travelers, which is probably underestimated—including only those who sought medical care from healthcare facilities throughout Thailand [5]. Common health problems among travelers include diarrhea, dermatological conditions, respiratory tract infections, injuries, fever and abdominal pain [3,5,7]. Increased travel to countries with endemic diseases like rabies significantly raised the number of travelers requiring post-exposure prophylaxis at GeoSentinel clinics by fourfold from 2003 to 2012 [8]. Regarding in-flight medical emergencies, which are particularly challenging due to the limited medical resources on board, an incidence of approximately 1 emergency per 604 flights was reported [9].

Cruise tourism also presents several health risks, particularly when medical emergencies exceed onboard handling capacity. Outbreaks such as food poisoning can strain both onboard health services and local medical facilities at ports [10]. Elderly travelers, often managing chronic illnesses or multiple health comorbidities, require special attention due to their increased vulnerability to health complications [11]. Although fatal events during travel are rare, estimated at approximately 1 in 100,000 travelers, they remain a significant concern [12,13].

Findings from previous literature reviews (one systematic and one narrative review) have demonstrated that seeking unplanned healthcare in foreign countries or unfamiliar locations presents several challenges for individuals [14,15]. Language difficulties and limited interpretation services are common barriers for tourists, often preventing them from fully understanding their health condition or financial responsibilities related to treatment. Cultural differences can also add to anxiety and confusion, as visitors unfamiliar with the structure and functioning of local healthcare systems may experience delays in receiving care. Insurance coverage and payments issues can also arise, sometimes leading to significant unpaid medical expenses. Additionally, limited access to patients’ medical histories can result in diagnostic errors, treatment delays and unnecessary repetition of medical procedures. Challenges in continuity of care are also present, as receiving appropriate follow-up care after an emergency abroad can be difficult, potentially affecting the patient’s recovery.

Countries with challenging geography such as Greece [16] often face difficulties in providing emergency healthcare in remote areas. Some key issues are the significant shortage of formally trained emergency medicine professionals and the unequal distribution of healthcare providers, especially on smaller islands and other rural locations. Healthcare services in such areas may only be available from a newly graduated physician or a general practitioner [17]. Consequently, patients with acute conditions often require air transportation to hospitals, significantly increasing costs. Although these flights typically have a short duration, considerable delays can occur due to weather conditions [17]. The situation is further complicated by Greece’s popularity as a tourist destination, contributing to considerable seasonal population fluctuations.

Growth in tourism may negatively impact the provision of local healthcare services, especially during peak seasons, limiting access to care for both tourists and local residents [18,19]. Emergency departments, in particular, face added pressures from handling non-urgent cases among tourists, potentially compromising the efficiency and speed of care delivery [18,19]. Importantly, many travel-related health problems can be mitigated through pre-travel consultations [7]. Furthermore, unexpected health situations can be managed more effectively if travelers are informed in advance about local healthcare resources, emergency procedures and important contacts [20]. However, pre-travel health consultations remain frequently neglected by international travelers. A study conducted by LaRocque et al. at Boston Logan International Airport (2010) [21] reported that nearly half of travelers heading to low- or low–middle-income countries did not seek any pre-travel advice.

Healthcare reimbursement is another critical issue. While European tourists typically utilize the European Health Insurance Card (EHIC) introduced in 2003 to streamline coverage [22], visitors from non-European countries often encounter administrative and insurance-related difficulties when accessing healthcare. Beyond these immediate challenges, socioeconomic factors also matter, as unexpected health problems may extend tourists’ stays abroad [5] and substantially increase their expenses.

This systematic review aims to summarize the current evidence regarding barriers that tourists face when seeking urgent medical care, as well as shedding light on the broader challenges posed by increased tourism on local healthcare systems. The ultimate goal of the current literature review is to contribute to redesigning tourism and health policy so that tourist destinations, in addition to being attractive and interesting, also become safer both for visitors and locals.

## 2. Materials and Methods

### 2.1. Design

A systematic review was conducted following the PRISMA guidelines, aiming to synthesize the current literature on barriers faced by tourists when accessing emergency healthcare services. The review protocol was registered with PROSPERO (ID CRD420251156975).

### 2.2. Search Strategy

In order to formulate a clear research question, the authors used the PICO model: patient or problem, intervention, comparison and outcome. In this study, the research question comprised three elements—PIO. Specifically (P) referred to tourists traveling to other regions; (I) the use of emergency healthcare; and (O) barriers, unmet healthcare needs and accessibility problems.

To address the research question, a literature search was conducted in the PubMed, Scopus, Web of Science and ScienceDirect databases, using the following search string: (tourist* OR traveler* OR visitor* OR tourism OR traveling OR visiting) AND (“healthcare services” OR “healthcare” OR “health care” OR “medical services” OR “seek* healthcare” OR “health services” OR “emergency medical care” OR “urgent health care” OR “emergency department visits” OR “rescue services”) AND (barrier* OR obstacle* OR challenge* OR limitation* OR issue* OR problem* OR delay* OR disparities* OR “unmet healthcare needs” OR inequality* OR “access” OR accessibility OR “healthcare availability” OR experiences OR “clinical outcomes” OR satisfaction).

The literature search was conducted from the end of September 2025 to the end of November 2025 using a title-specific search criterion, ensuring that only studies with a primary focus on the topic were retrieved. This title-based search approach was employed to increase the specificity and relevance of the articles assessed, to reduce false positive selections and finally to ensure a manageable number of articles. An additional filter was applied to include only articles published after the year 1995, aiming to capture literature from the last 30 years. Moreover, backward/forward citation searching was conducted.

Two independent researchers (P.P. and D.A.) screened all identified records for eligibility. The article selection procedure began with title screening, followed by abstract review, and concluded with full-text assessment for eligibility. Inter-rater agreement between the two independent researchers was reported for the majority of articles. However, any uncertainties regarding article inclusion were resolved through discussion with the principal investigator.

### 2.3. Inclusion/Exclusion Criteria

The inclusion criteria for this review were carefully defined to ensure the relevance of the selected studies. First of all, eligible studies had to focus on tourists (either national/domestic or international), with main purposes of travel, leisure, business, education, adventure and cultural experience. Therefore, studies focusing on travelers such as migrants, expatriates or routine cross-border visitors were excluded.

Secondly, the health services sought by these tourists had to be unplanned or emergency in nature, reflecting acute healthcare needs rather than scheduled medical interventions. Thirdly, only studies published in English or Greek were considered in order to align with the language capabilities of the research team. Finally, only studies published after 1995 were included to ensure that the review captured contemporary challenges in the field of tourist healthcare. However, studies were excluded if they focused primarily on medical tourism, spa or wellness tourism, planned healthcare interventions or traveling communities seeking routine care.

### 2.4. Study Selection and Outcome

A comprehensive search was performed in PubMed, Scopus, Web of Science and ScienceDirect from 1st January 1995 to 31 October 2025.

A total of 603 articles were identified through database searches, along with 5 additional studies found via reference list screening and supplementary searches of related articles. After removing 108 duplicate records, 500 unique entries remained. Following initial screening of titles and abstracts, 481 records were excluded, resulting in 19 potentially relevant articles; these were then independently assessed (by two researchers) for eligibility, and 9 records were excluded as they did not meet the inclusion criteria.

**Figure 1:**
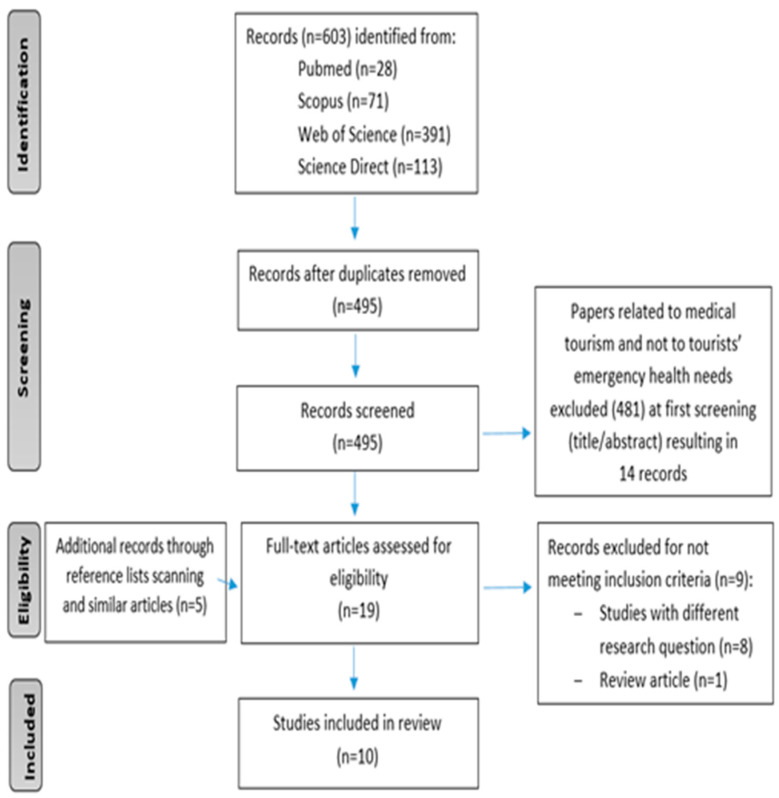
The study selection process based on PRISMA flowchart.

As a result, 10 studies were included in the final systematic review. The study selection process is illustrated in the PRISMA flowchart shown in Figure 1.

**Figure 2:**
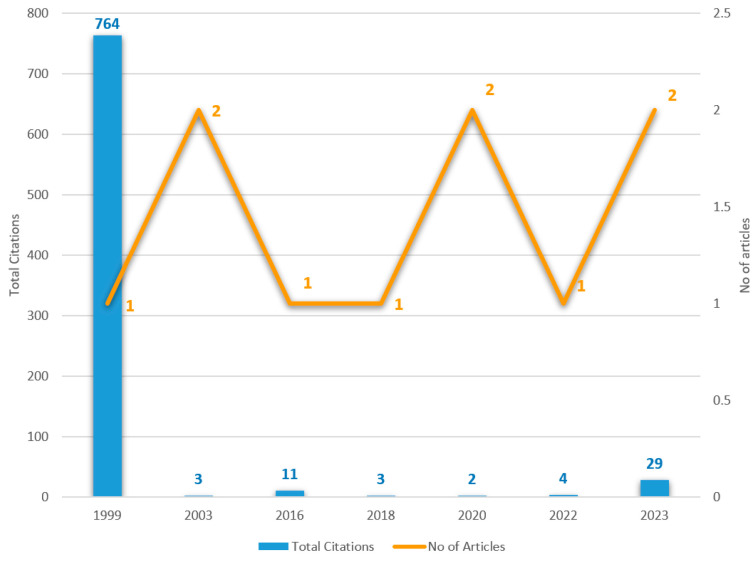
Evaluation of articles and citations (total citations of all articles published per year).

Moreover, Figure 2 presents the citation evolution of the reviewed articles.

### 2.5. Data Analysis and Synthesis

The following information was extracted from each study: year of publication, study location, study duration, sample size, sample characteristics (mean/median age, gender, and nationality), study type and key findings. Additionally, the methodological quality of each included study was assessed (see the following paragraph). A meta-analysis was not possible due to the methodological heterogeneity of the included studies (cross-sectional studies were analyzed along with retrospective reviews of medical records); therefore, a narrative synthesis of the results was conducted.

The results are organized based on the healthcare ***access model*** of Penchansky and Thomas [23]. Based on the National Academies of Sciences, Engineering and Medicine, access to healthcare is defined as *“timely use of personal health services to achieve **the best possible health outcomes”**.* However, some patients face barriers that limit access to necessary healthcare, creating inequalities that can potentially adversely affect health outcomes [24]. Therefore, it is necessary to analyze the distinct dimensions that make up the concept of access to healthcare and their connected impact on patients’ and health systems’ outcomes [24,25].

The accessibility dimensions are defined as follows [23]:

- ***Availability*** related to sufficiency of health services and professionals.
- ***Accessibility*** focused on geographic distance and time to access health services.
- ***Acceptability*** referring to match between patients’ and providers’ attitudes (including cultural and social norms).
- ***Affordability*** related to patients’ ability to pay for health services.
- ***Adequacy*** focused on effective and appropriate response to patients’ needs.

### 2.6. Quality Assessment

The quality assessment of the included studies was conducted using the Quality Assessment Tool for Observational Cohort and Cross-Sectional Studies, developed by the National Institutes of Health [26]. This tool consists of 14 specific questions and provides a structured and consistent framework for identifying potential biases, evaluating the internal validity of each study and assessing the overall methodological quality of each study. It examines key elements such as the clarity of the research question, selection and definition of the study population, measurement of exposures and outcomes and handling of confounding variables. There are three possible answers: “yes”, “no” and “other” (i.e., cannot determine, not applicable or not reported). Based on the responses, each study is assigned a quality rating of either good, fair or poor, reflecting its overall risk of bias.

In general, five studies were assessed as good or fair, and key conclusions reported in the current literature review are based on these. Three could not be assigned a quality rating (due to limited information) and two were assessed as poor. In all reviewed studies, both the research question or objective and the population were clearly specified and defined. Moreover, in seven out of ten studies the outcome measures (dependent variables) were clearly defined, valid, reliable and implemented consistently across all study participants (while in the other three studies, this was not applicable). Finally, the exposure measures (independent variables) were clearly defined and implemented consistently across all study participants in six out of ten studies.

## 3. Results

### Characteristics of the Studies

This review included 10 quantitative studies, of which 5 used a cross-sectional design and 5 a retrospective design. All studies were published between 1999 and 2023. Most (7/10) were conducted in Asian countries (Japan, Turkey, Saudi Arabia and Thailand), while the remaining three were conducted in Europe [27], the U.S.A. [28] and multiple countries [7]. The number of participants ranged from 37 to 2333. All studies included both genders, except for one that focused exclusively on obstetrical emergencies in pregnant women [29] (Table 1). Common health issues included gastrointestinal problems (diarrhea and abdominal pain), injuries, infectious diseases (dengue, malaria and rabies exposure) and pregnancy-related emergencies.

**Figure 3:**
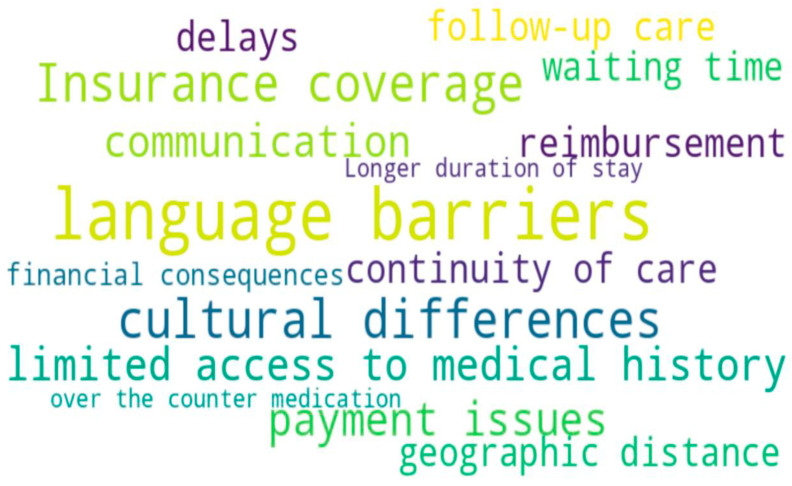
The most common words of abstracts describing barriers.

Figure 3 summarizes the most frequently reported barriers faced by tourists seeking emergency care.

Moreover, in Table 2, the results are classified based on the Penchansky and Thomas [23] framework.

**Acceptability** issues related to language, cultural and religious differences, as well as issues related to communication with the healthcare providers (e.g., unanswered questions), proved to be the most frequently reported difficulties on behalf of tourists [28,29,31,32,33]. Tourists often reported difficulties in understanding healthcare staff and insufficient explanations regarding their condition or treatment [3]. A large proportion of them also reported that they needed professional interpretation services (up to 54%) [31,33], but in some cases there was a complete lack of access [28]. Even when interpretation was provided, interpreters usually lacked formal training [28] or they had limited specialization in different languages [33]. In addition, based on the results of Taguchi et al.’s study [32] conducted in Japan, some issues arose due to the lack of consideration for religious customs and the location where the tourist wished to receive treatment. Consistent with previous results, Nakazawa et al. [29] found that two of the main challenges tourists faced were language and cultural barriers.

In addition, **financial consequences and insurance coverage** issues (related to **affordability**) were investigated in three out of ten articles. A study conducted in two German travel clinics by Saffar et al. [27] identified that one in three participants (32%) had to spend more than €100 after potential rabies exposure. Interestingly, almost half of the participating travelers (48%) were unaware at the time of the study whether their costs could be reimbursed by their health insurance. Nakazawa et al. [29] similarly reported that differences in health insurance systems were among the main challenges faced by tourists. In another study by Mansanguan et al. [5] on adult backpackers who visited Thailand, one in five participants with health problems (20%) reported that they had to buy over-the-counter medication.

Moreover, **availability** and **accessibility** barriers were also identified. A study conducted in Saudi Arabia by Alsharif A.I. and Al-Khaldi Y.M [30] suggested that participants without Saudi nationality, compared to Saudis, indicated that the primary healthcare centers’ working hours were inappropriate. Approximately one in four tourists (22.4%) in the same study suggested establishing a 24-h duty service. The accessibility of emergency healthcare services was also problematic for tourists after a possible rabies exposure [27]. In this study, approximately half of the participants (52%) reported being able to reach a healthcare facility within one hour, while almost all (92%) could do so within 24 h, highlighting possible geographical constraints and reduced availability of healthcare facilities.

Finally, **adequacy** problems were also identified. Saffar et al. [27] found that one in five individuals who sought medical care abroad (20%) did not receive any vaccination, despite its necessity, highlighting that tourists did not receive the appropriate care abroad. Among those who were vaccinated and provided detailed information, about one in four (26%) received the vaccine only after visiting at least a second healthcare facility. Furthermore, Piyaphanee et al. [7] conducted a study using data from travelers evaluated at GeoSentinel network sites, which are specialized travel and tropical medicine clinics in 28 countries. The investigators suggested that travelers were more likely to receive complete reports on their trip-related healthcare exposure when evaluated at GeoSentinel clinics after returning home (92%), rather than during travel, emphasizing gaps in the continuity of care and quality of medical documentation during travel.

## 4. Discussion

This systematic review highlights that tourists seeking emergency healthcare abroad may face significant challenges impacting both the quality of care received and their overall travel, as well as the health system.

### 4.1. Impact on Patient

**Language barriers** proved to be the biggest obstacle in patient–provider communication [28,29,31,32,33]. As a result, some patients were unable to communicate with medical staff. Carrasquillo et al. [28] investigated the impact of language differences on patients’ experience in the emergency department and found that only half of non-English-speaking patients (52%) experienced satisfaction with the care provided, compared with 71% of English-speaking patients. Specifically, non-English-speaking patients were more likely to experience difficulties with communication, diagnostic testing, the use of medication and follow-up care, ultimately **reducing the overall quality of healthcare.**

**Insurance and payment-related issues are also substantial challenges**, especially for uninsured patients [10,15]. Limited or absent coverage and generally complex reimbursement procedures can delay access to necessary treatment. In emergency settings, where prompt intervention is critical, any delays caused by insurance verification may compromise patient outcomes. These challenges can be particularly important for tourists requiring **extended hospitalization, as prolonged stays significantly increase total travel costs** [10,15], e.g., due to the cancelation of return tickets and purchase of new ones.

**Cooperation with external organizations** such as embassies or transportation services was also reported as challenging, further complicating the patient journey [33]. Τhese barriers may also have indirect consequences on overall tourists’ travel experience, as health problems frequently prolonged the duration of stay abroad and led to postponements of planned activities or, in some cases, cancelations [5,27]. Such outcomes not only reflect the medical burden, but also highlight the social and economic implications of seeking emergency care while traveling.

### 4.2. Impact on Health System

**Language and cultural differences** can also be **critical barriers** to the delivery of **efficient emergency care**. Communication difficulties and cultural differences can also lead to delays in decision-making, reduced adherence to care plans and **additional strain on already limited ED resources** [15]. Moreover, language barriers were linked to **longer emergency department stays** (up to approximately 50 min) [31] and in some cases **to unpaid medical bills** due to tourists misunderstanding [32].

**Limited access to a patient’s medical history** is also a significant challenge during emergency visits, hindering both diagnostic accuracy and **treatment efficiency**. When healthcare providers are unable to receive essential clinical information, informed decision-making is difficult and the provision of care may be delayed. The absence of medical records may also lead to repetition of diagnostic tests, driving up **healthcare costs and extending ED length of stay** [10,15].

**Emergency departments (EDs) face significant challenges during peak vacation periods** [18,19,34,35]. EDs in tourist areas often lack the resources to handle the high number of admissions, a portion of which are non-urgent [19,36]. Especially during the summer, when staff availability may be reduced due to their scheduled vacations, increased tourism can overwhelm existing resources and influence treatment speed, leading to prolonged wait times, delays in diagnosis and an overall decline in the quality of care [10,15,18,19,36]. This can be particularly important in geographically isolated or resource-limited settings, such as islands. A study conducted in Corfu (Greece, 2022) indicated that injury admissions during peak tourist season (summer months) have led to more frequent patient transfers to other healthcare facilities, particularly when injuries occur outside the hospital’s full working schedule [34]. Similar patterns have been observed in winter sports destinations like Switzerland, where hospital admission rates with orthopedic patients can be up to four times higher in winter compared to other seasons, largely due to non-local residents [35]. Another study conducted in Sorrento (Italy, 2021) indicated that tourists may receive faster ED treatment than local residents, potentially limiting their timely access to emergency care [18]. Additionally, unpredicted events such as mass food poisoning on cruise ships can sharply increase demand on the nearest healthcare facilities, placing additional stress on local health systems [10].

### 4.3. Limitations

One of the primary limitations of this study is the inclusion of literature published exclusively in English and Greek, which may have resulted in the exclusion of relevant studies available in other languages. Moreover, this language restriction may raise bias issues toward destinations and health systems more represented in English language publishing. Furthermore, by limiting the search to publications after 1995, earlier and potentially significant literature might have been overlooked. On the other hand, searching for papers published in previous decades led to the inclusion (in the current literature) of a few articles considered as old. Therefore, some of the barriers mentioned in these articles may have been mitigated, for example, through the use of telemedicine and translation applications. Although efforts were made to identify comprehensive and relevant sources, gray literature was not systematically explored; this raises the possibility that important data may have been missed. In addition, the initial search was conducted based on titles, resulting in a manageable number of 608 articles. However, it is recognized that a title-based search is likely to miss a few relevant studies.

Another notable limitation is the absence of a meta-analysis. This decision was based on the considerable methodological heterogeneity among the included studies, which made quantitative synthesis unfeasible. As a result, the strength and generalizability of the study’s conclusions are limited and there is a greater dependence on qualitative interpretation, which carries a degree of subjectivity.

In addition, the findings may be influenced by publication bias, where studies with positive or statistically significant outcomes are more likely to be published, while those with negative results remain unpublished, potentially affecting the representativeness and accuracy of the overall conclusions. Lastly, database selection bias may have played a role, as the search was confined to four major databases, potentially excluding relevant studies published in less prominent or regional repositories.

## 5. Conclusions and Recommendations

Based on the evidence to date (given that the literature search was completed in November 2025) and taking into consideration the limited number (n = 10) of specialized manuscripts focused on an emerging nature topic, which come mainly from Asia, we can conclude that tourists face multiple barriers when seeking emergency healthcare abroad: linguistic, financial, organizational and cultural. Addressing these issues requires systemic changes, while several targeted strategies can be implemented before, during and after travel to mitigate the aforementioned impacts at both the patient and health system levels.

Pre-Travel Recommendations: Preparation plays a critical role in reducing travel-related health risks. Tourists should have a pre-travel medical consultation, especially those traveling to developing countries or those with chronic illnesses [11,27]. They should be informed according to the destination and the length of their trip about preventive strategies such as necessary vaccinations, guidance on managing potential health issues and advice on when and how to seek and access timely medical help in case of emergency [11]. Travelers with known health conditions are recommended to obtain a letter of referral from their personal physician before departure [20]. Moreover, tourists should be encouraged to prepare a comprehensive health record with their updated medical information such as vaccination certifications, potential allergies, blood type, insurance and emergency contact information [11]. They should also conduct appropriate research regarding the available health services and the proximity of medical facilities. For individuals who take regular medication, renewing prescriptions and ensuring an adequate supply is essential [20].

Recommendations During Travel: In case of facing a language barrier, using professional interpretation services or designing an app for interpretation can help to reduce communication difficulties and ensure proper treatment [18,37]. Understanding the local emergency system is also essential. Tourists should learn the emergency telephone number of the host country as soon as they arrive, or ideally before departure [20]. Furthermore, the availability of ambulance services may vary widely between urban and rural areas. Knowing the transportation availability and the distance to the nearest healthcare facility is very important, especially for families traveling with children or those traveling to areas with limited emergency care. In such cases, tourists are also advised to carry a basic first aid kit and essential over-the-counter medications, and even be prepared to apply basic emergency procedures like CPR [20]. In regard to highly visited tourist areas, particularly during peak seasons, increasing healthcare staff availability becomes crucial [3,10].

Moreover, in order to mitigate financial uncertainty and reimbursement confusion, destinations and insurers can co-develop standardized tourist billing explanations and upfront cost disclosure protocols.

Post-Travel Recommendations: Even after returning home, continuity of care remains important. Tourists should be given a detailed summary or referral letter from the foreign healthcare facility they have visited. This document should include the medical interventions performed and provide recommendations for follow-up care [11].

## Figures and Tables

**Table 1:** Analysis of the studies included.

REGION	Reference/Year/Country	Data Collection Time	Sample Size (n) Sex (%)	Age	Tourist Nationality	Study Design Sampling Method	RESULTS—BARRIERS
**EUROPE**	Saffar et al., 2023 [27] Germany	March 2019 to June 2020	75 59% females	Median (IQR): 34 years (26–43)	Region of exposure (n = 72) Asia 47.2% Africa 22.2% Europa 19.4% Latin America and Caribbean 5.6% Middle East 5.6% Region of birth (n = 75) Germany 82.7% Europe (Germany excluded) 9.3% Other 8.0% Region of residence (n = 75) Germany 97.3% Europe (Germany excluded) 2.7%	Cross-sectional Convenience sample	About one in five individuals who sought medical care abroad (20%) did not receive any vaccination. Among those who were vaccinated and provided detailed information, about one in four (26%) received the vaccine only after visiting at least a second healthcare facility. Approximately half of the participants (52%) reported being able to reach a healthcare facility within one hour, while almost all (92%) could do so within 24 h. Among the 44 travelers who reported the impact of potential rabies exposure on their journey, 5% canceled their trip while 7% modified their travel plans following the suspected exposure. About one in three participants (32%) who provided information on the financial consequences after their potential rabies exposure reported spending more than 100 euros. Almost half of the participants (48%) were unaware at the time of the study whether their costs could be reimbursed by their insurance.
**USA**	Carrasquillo et al., 1999 [28] United States of America (Northeastern region)	February to May 1995	2333 86% males 42% English speakers	Mean (sd): English Speakers 47 years (20) non-English speakers 41 years (18)	Race of English speakers White 78% Black 16% Latino 3% Asian/Other 3% Race of non-English speakers White 24% Black 11% Latino 50% Asian/Other 15%	Cross-sectional Convenience sample	Only half of non-English-speaking patients (52%) were satisfied with the care provided in the emergency department (ED), compared with 71% of English-speaking patients. Across all participants, patient satisfaction rates ranged from 56% to 81% (*p* < 0.01). Interpretation services were not commonly provided in the ED and, when provided, interpreters usually lacked formal training and necessary skills. Non-English-speaking patients were significantly more likely to present with less-urgent problems, lack a primary care provider, rely on the ED as their main source of care, have no health insurance and have Medicaid coverage. 14% of non-English speakers indicated they would avoid returning to the same ED in case of a future emergency, compared with 9.5% of English speakers (*p* < 0.05). Non-English-speaking patients were more likely to experience difficulties with communication (*p* < 0.001), diagnostic testing (*p* < 0.01), the use of medication (*p* < 0.01) and follow-up care (*p* < 0.06).
**EUROPE-ASIA**	Tekin, 2003 [3] Turkey (Marmaris)	7 January 2002 to 9 February 2002	413 52.5% males	% of age categories: 31.5% 30–39 years 31.0% 20–29 years 14.8% 40–49 years 10.9% 10–19 years 11.9% ≥50 years	USA 12.3% German 11.6% French 11.6% Russian 11.4% Dutch 11.4% Swiss 10.9% Belgian 10.7% British 10.4% Danish 9.7%	Cross-sectional Convenience sample	13.5% of participants reported difficulties in communication. Approximately one in ten participants (10.8%) indicated that healthcare staff did not provide sufficient explanations Almost one in four participants (22.8%) reported that providing information to foreign visitors would be beneficial.
	Piyaphanee et al., 2023 [7] The GeoSentinel network clinics in 28 countries	May 2017 to June 2020	1093 55% females	Median: 36 years (range: 1–94)	Top destinations where travelers received unplanned healthcare India (10%) Thailand (5%) Mexico (4%) Indonesia (3%) Philippines (2%) Romania (2%) Vietnam (2%) Tanzania (2%) Dominican Republic (2%) Cambodia (2%)	Cross-sectional Convenience sample	After receiving medical care abroad, 16% of the patients reported that their health problem was completely resolved, 59% noted improvement, 21% experienced no change and few patients (3%) indicated that their condition subsequently worsened. Travelers were more likely to receive complete reports on their trip-related healthcare exposure when evaluated at GeoSentinel sites after returning home (92%), rather than during travel (8%).
**ASIA**	Alsharif A.I. & Al-Khaldi, Y.M., 2003 [30] Saudi Arabia (Aseer region)	July of 2000	413 81.4% males	Mean (sd): 29.2 years (13.9)	Nationality Saudi 81.4% Non-Saudi 10.2% Unknown 4.1% Place of residence: Western region 32.7% Central region 22% Southern region 18% Eastern region 6.3% Gulf states 9% Unknown 12%	Cross-sectional Convenience sample	A small proportion of tourists expressed dissatisfaction with the consultation and treatment process, as 12% stated that physicians had not answered their questions, and 14% that no physical examination had been performed. In addition, one in five (19%) indicated that nurses had not taken their vital signs. About one in five participants (21.5%) reported not receiving instructions on their medications by pharmacists. Participants without Saudi nationality, compared to Saudis (*p* = 0.02), indicated that the PHCC working hours were inappropriate. Approximately one in four tourists (22.4%) suggested establishing a 24-h duty service. Around one in six (16.4%) recommended increasing the availability of female doctors. 15.5% proposed the installation of guidance signboards. Nearly one in seven (13.8%) suggested the extension of PHCC. 12.0% of the participants would have liked an increase in staff.
	Mansanguan et al., 2016 [5] Thailand (Bangkok)	May to July 2015	420 51.4% males	Median: 26.4 years (range: 18–68)	European 66.9% North American 17.4% Asian 10.5% Australian and New Zealander 2.9% Latin American 1.9% African 0.5%	Cross-sectional Convenience sample	Participants with health problems had a significant longer mean duration of stay than participants without health problems (23.1 vs. 8.8 days, *p* < 0.001). One in five backpackers with health problems (20%) reported that they had to buy over-the-counter medication. Nearly one in four travelers with health problems (23.3%) had to delay or postpone their trip for at least one day, while 4.7% had to cancel a planned trip or activity.
	Aoki et al., 2022 [31] Japan (Negano)	1 April 2018 to 31 March 2020	777 40.7% males	Median (IQR): 37 years (24–50)	Chinese: 19.6% Filipino: 13.4% Brazilian: 9.3% Australian: 5.0% Korean: 5.0% Thai: 4.6% Vietnamese: 4.1%	Retrospective review of medical records	About one fifth of the patients (20.7%) needed medical interpretation by hospital staff. 5.8% of the patients had interpretation by their family members. Approximately one in six patients (17.6%) received translation by accompanying persons. A small portion of patients used phone interpreters (0.4%) or applications (0.4%) for translation. Speaking a language other than Japanese, Chinese or English was linked to an additional emergency department length of stay (EDLOS) of 51.7 min (95% CI, 17.8–85.6). Additional factors were found to extend patients’ length of stay in the emergency department. These included the nature of medical investigations and treatments carried out, procedures performed within the ED, and the need for consultation with a specialist.
	Taguchi et al., 2018 [32] Japan (Hokkaido)	April 2012–March 2016	132 46.2% males	Mean: 40 years (range 2–97)	Chinese: 27% Other: 73%	Retrospective review of medical records	Language barriers proved to be the biggest obstacle in patient–provider communication. The hospital in which this study was conducted was not adequately prepared to communicate with Chinese-speaking patients. Difficulties in communication led to unpaid medical bills in some cases. Cultural and religious differences between patients and providers resulted in disagreements. For example, some issues arose due to the lack of consideration for religious customs and the location where the tourist wished to receive treatment.
	Nakazawa et al., 2020 [29] Japan (Okinawa)	January 2014 to December 2018	37 100% females	Not reported	Countries of origin China: 10 cases Taiwan: 9 cases Korea: 9 cases Hong Kong: 4 cases Australia: 2 cases Canada: 2 cases Brazil: 1 case	Retrospective review of medical records	The main challenges for tourists were language and cultural barriers, as well as differences in health insurance systems.
	Shimoyama et al., 2020 [33] Japan (Tokyo)	2020	87 66.7% males	Median (IQR): 36 years (24–56)	Race Asian 74% (Chinese 33%) Other 26%	Retrospective review of medical records	Approximately half of the patients (54%) needed professional interpretation services. Nearly 15% of the patients had their medical bills unpaid. Reported challenges included difficulties in cooperating with other organizations (e.g., each country’s embassy and international transportation services). Language and cultural differences led to difficulties in communication and understanding between patients and healthcare professionals in a few cases. As a result of the interpreters’ limited specialization in different languages, some patients were unable to communicate with the medical staff.

**Table 2:** Accessibility barriers and impact on patients’ and health systems’ outcomes based on the studies included on the current literature review.

Dimension of Accessibility/Main Issues Reported per Author	Authors/Year/(Country)	Impact on Patients	Impact on Health System
**Availability** **I.** Insufficient working hours of PHCC and insufficient staff members	**I.** Alsharif A.I. & Al-Khaldi, Y.M., 2003 (Saudi Arabia) [30]	**I.** NR	**I.** NR **II**. NR
**Accessibility** I. NR II. About half of the participants being able to reach a healthcare facility within 1 h, and the vast majority able to do so within 24 h	**I.** Mansanguan C. et al., 2016 Thailand [5] **II.** Saffar F. et al., 2023 (Germany) [27]	**II** & **III.** Longer mean duration of stay and postponement or cancelation of a trip or activity for a minority of the patients	
**Acceptability** **I**. Communication barriers and insufficient explanations by healthcare staff **II**. Unanswered questions on behalf of physicians, no instructions for medications by the pharmacists **III & IV & V & VII.** Language/cultural/religious barriers and lack of or limited interpretation services	**I.** Tekin Y., 2003 Turkey (Marmaris) [3] **II.** Alsharif A.I. & Al-Khaldi, Y.M., 2003 (Saudi Arabia) [30] **III.** Nakazawa T. et al., 2020 (Japan) [29] **IV.** Carrasquillo O. et al., 1999 (USA) [28] **V**. Aoki Y. et al., 2022 (Japan) [31] **VI.** Taguchi D. et al., 2018 (Japan) [32] **VII.** Shimoyama K. et al., 2020 (Japan) [33]	**IV.** Lower satisfaction from health system for non-English speakers **V. A**dditional emergency department length of stay => patient hassle **VI**. Lack of consideration for religious customs and the location where the tourist wished to receive treatment **VII.** Difficulties in understanding between patients and healthcare professionals in a few cases and difficulties in cooperating with other organizations (e.g., country’s embassy)	**IV.** Non-English-speaking patients more likely to present with less-urgent problems and use the ED as their main source of care, resulting in overcrowded EDs **V.** Additional emergency department length of stay leading to overcrowded EDs **VI &VII.** Unpaid medical bills in some cases
**Affordability** **II.** Almost half of participants were unaware whether costs could be reimbursed by insurance **III**. Differences in health insurance systems	**I**. Mansanguan C. et al., 2016 (Thailand) [5] **II.** Saffar F. et al., 2023 (Germany) [27] **III.** Nakazawa T. et al., 2020 (Japan) [29]	**I.** Need to buy over-the-counter medication	**I**. NR
**Adequacy** **Ι.** About 20% who sought medical care abroad did not receive any vaccination, although they were in need. Among those who were vaccinated, 26% received the vaccine only after visiting at least a second healthcare facility **II**. No physical examination performed by physicians and no vital signs taken by nurses **III.** NR	**I.** Saffar F. et al., 2023 (Germany) [27] **II**. Alsharif A.I. & Al-Khaldi, Y.M., 2003 (Saudi Arabia) [30] **III.** Piyaphanee W. et al., 2023 (GeoSentinel network clinics in 28 countries) [7]	**I.** **II. NR** **III.** 3 out of 4 patients reported that their health problem was completely resolved, and 59% noted improvement after receiving medical care abroad	**I & II & III. NR**

NR: Not reported.

## Data Availability

The original contributions presented in this study are included in the article/Supplementary Materials. Further inquiries can be directed to the corresponding author(s).

## Supplementary Materials

The following supporting information can be downloaded at: https://www.mdpi.com/article/10.3390/healthcare14060761/s1.
