# Supplementary material for: Investigating Tourists’ Emergency Healthcare Access Barriers: A Systematic Literature Review
Source: Healthcare (Basel). 2026 Mar 18;14(6):761. doi: 10.3390/healthcare14060761 (PMC13026848; doi:10.3390/healthcare14060761)
Supplement: Supplementary file 1 [file healthcare-14-00761-s001.zip › healthcare-4095961-supplementary.pdf]

	Criteria	Tekin Y., 2003 [3]	Alsharif A.I. & Al-Khaldi, Y.M., 2003 [30]	Mansanguan C. et al, 2016 [5]	Piyaphanee W. et al, 2023 [7]	Saffar F. et al., 2023 [27]	Carrasquillo O. et al, 1999 [28]	Aoki Y. et al, 2022 [31]	Shimoyama K. et al, 2020 [33]	Taguchi D. et al, 2018 [32]	Nakazawa T. et al, 2020 [29]
1	Was the research question or objective in this paper clearly stated?	YES	YES	YES	YES	YES	YES	YES	YES	YES	YES
2	Was the study population clearly Specified and defined?	YES	YES	YES	YES	YES	YES	YES	YES	YES	YES
3	Was the participation rate of eligible persons at least 50%?	NOT REPORTE D	YES	NOT REPORTED	NOT REPORTED	YES	YES	YES	NOT APPLICABLE	NOT APPLICABLE	NOT APPLICABLE
4	Were all the subjects selected or recruited from the same or similar populations (including the same time period)? Were inclusion and exclusion criteria for being in the study prespecified and applied uniformly to all participants?	YES	YES	YES	YES	YES	NO	YES	NOT APPLICABLE	NOT APPLICABLE	YES
5	Was a sample size justification, power description, or variance and effect estimates provided?	NO	NO	YES	NO	NO	NO	NO	NOT APPLICABLE	NOT APPLICABLE	NOT APPLICABLE
6	For the analyses in this paper, were the	NO<br><br>Cross	NO<br><br>Cross	NO<br><br>Cross sectional	NO<br><br>Cross sectional	NO<br><br>Cross	NO<br><br>Cross sectional	YES	NOT APPLICABLE	NOT APPLICABLE	NOT APPLICABLE

	exposure(s) of interest measured prior to the outcome(s) being measured?	sectional design	sectional design	design	design	sectional design	design				
7	Was the time frame sufficient so that one could reasonably expect to see an association between exposure and outcome if it existed?	NO	NO	NO	NO	NO	NO<br><br>Cross sectional design	YES	NOT APPLICABLE	YES	YES
8	For exposures that can vary in amount Or level, did the study examine different levels of the exposure as related to the outcome (e.g., categories of exposure, or Exposure measured as continuous variable)?	NOT APPLICABLE	NOT APPLICABLE	NOT APPLICABLE	NOT APPLICABLE	YES<br><br>Some travelers had pretravel consultation, or pre travel vaccination	NOT APPLICABLE	YES	NOT APPLICABLE	NOT APPLICABLE	NOT APPLICABLE
9	Were the exposure measures (independent variables) clearly defined, valid, reliable, and implemented consistently across all study participants?	YES	YES	YES	YES	YES	YES	NO	NOT APPLICABLE	NOT APPLICABLE	NOT APPLICABLE
10	Was the exposure(s) assessed more than once	NO	NO	NO	NO	NO	NO	NO	NOT APPLICABLE	NOT APPLICABLE	NOT APPLICABLE

	overtime?										
11	Were the outcome measures (dependent variables) clearly defined, valid, reliable, and implemented consistently across all study participants?	YES	YES	YES	YES	YES	YES	YES	NOT APPLICABLE	NOT APPLICABLE	NOT APPLICABLE
12	Were the outcome assessors blinded to the exposure status of participants?	NOT APPLICABLE	NOT APPLICABLE<br><br>self-reported questionnaire	NOT APPLICABLE<br><br>self-reported questionnaire	NOT APPLICABLE	NOT APPLICABLE<br><br>self-reported questionnaire	NOT APPLICABLE	NO	NOT APPLICABLE	NOT APPLICABLE	NOT APPLICABLE
13	Was loss to follow-up after baseline 20% or less?	NOT APPLICABLE	NOT APPLICABLE	NOT APPLICABLE	NOT APPLICABLE	NOT APPLICABLE	YES	NO	NOT APPLICABLE	NOT APPLICABLE	NOT APPLICABLE
14	Were key potential confounding variables measured and adjusted statistically for their impact on the relationship between exposure(s) and outcome(s)?	NO	NO	NO	NO<br><br>Only descriptive statistics	NO<br><br>Only descriptive statistics	NO	YES	NOT APPLICABLE	NOT APPLICABLE	NOT APPLICABLE
	**Evaluation**	**POOR**	**FAIR**	**FAIR**	**POOR**	**FAIR**	**FAIR**	**GOOD**	**IMPOSSIBLE TO BE ASSESSED**	**IMPOSSIBLE TO BE ASSESSED**	**IMPOSSIBLE TO BE ASSESSED**
